# An algorithm to predict advanced proximal colorectal neoplasia in Chinese asymptomatic population

**DOI:** 10.1038/srep46493

**Published:** 2017-04-18

**Authors:** Jason Liwen Huang, Ping Chen, Xiaoqin Yuan, Yunlin Wu, Harry Haoxiang Wang, Martin Chisang Wong

**Affiliations:** 1 JC School of Public Health and Primary Care, Faculty of Medicine, Chinese University of Hong Kong, Hong Kong SAR, China; 2 Ruijin Hospital North, Shanghai Jiaotong University, Shanghai 201801, China; 3 School of Public Health, Sun Yat-sen University, Guangzhou 510080, Guangdong, China; 4 Institute of Digestive Disease, Faculty of Medicine, the Chinese University of Hong Kong, Shatin, NT, Hong Kong SAR, The People’s Republic of China; 5 State Key Laboratory of Digestive Disease, Faculty of Medicine, Chinese University of Hong Kong, Shatin, NT, Hong Kong SAR, The People’s Republic of China.

## Abstract

This study aims to develop and validate a new algorithm that incorporates distal colonoscopic findings to predict advanced proximal neoplasia (APN) in a Chinese asymptomatic population. We collected age, gender, and colonoscopic findings from a prospectively performed colonoscopy study between 2013 and 2015 in a large hospital-based endoscopy unit in Shanghai, China. Eligible subjects were allocated to a derivation group (n = 3,889) and validation group (n = 1,944) by random sampling. A new index for APN and its cut-off level were evaluated from the derivation cohort by binary logistic regression. The model performance was tested in the validation cohort using area under the curve (AUC). Age, gender, and distal finding were found to be independent predictors of APN in the derivation cohort (p < 0.001). Subjects were categorized into Average Risk (AR) and High Risk (HR) based on a cut-off score of 2. The AUC of the derivation and validation cohorts were 0.801 (0.754–0.847) and 0.722 (0.649–0.794), respectively. In the validation cohort, those in the HR group had a 3.57 fold higher risk of APN when compared with the AR group (P < 0.001), requiring 18 (95% CI = 12–28) follow-up colonoscopies to detect 1 APN. This new clinical index is useful to stratify APN risk in Chinese population.

Worldwide, colorectal cancer (CRC) is the third most common cancer in males and ranks the second in females, with an estimated 1.4 million cases and 693,900 deaths reported in 2012^1^. The increasing incidence and mortality of CRC in many low-income and middle-income countries, including China, highlights the pressing need for CRC control^2^. Screening has been proven an effective and cost-efficient strategy to reduce CRC mortality^3^.

CRC prevention should be the primary goal of screening. Tests that are employed to detect early cancer and adenomatous polyps have been recommended for average-risk subjects aged 50 years or above^4^, and these include colonoscopy and flexible sigmoidoscopy (FS). In recent decades, proximal shift of CRC and advanced proximal neoplasia (APN) with no distal lesions detected^5^,^6^,^7^,^8^,^9^ are increasingly observed, and this has an implication on the use of FS in clinical practice. Nevertheless, colonoscopy might not be suited as a primary screening test in resource-limited countries with limited colonoscopy capacity^10^. It may be more cost-effective and cost-saving to reserve colonoscopy for subjects with high risk for APN in population-based screening^11^,^12^. To address this need, Imperiale *et al*.^12^ proposed a 7-point scoring system to predict APN by using age, gender, and distal findings at FS as predictors and a high discrimination value was reported in its internal validation group. Its generalizability remain unknown in Chinese subjects, since the incidence and distribution of colorectal neoplasia are different among different ethnic groups^13^. Levitzky *et al*. has performed an external validation of the model devised by Imperiale *et al*., and the discriminatory capability was found to be lower in a different population, including black and Hispanic^11^. to the primary objective of this study is to develop and validate a new model specifically tailored to predict APN in a Chinese asymptomatic population. We conducted this cross-sectional study in a large hospital-based endoscopy unit.

## Results

### Descriptive findings

Table 1 showed that the age, gender, and the most advanced distal findings of the derivation and validation cohorts were statistically similar (all p-values > 0.05). For the derivation cohort and validation cohort, the APN rates were 2.7% (95% CI, 2.2–3.2%) and 2.4% (95% CI, 1.8–3.2%), respectively. The Cochran Mantel–Haenszel χ^2^ test indicated that age, gender, and distal findings were associated with APN. The prevalence of APN in the derivation cohort stratified by these variables was shown in Table 2.

### Univariate and multivariate binary logistic regression of advanced proximal neoplasia in the derivation group

Univariate and multivariate analyses were performed for each risk factor. Multivariate binary logistic regression showed that age above 55 years, male gender, and distal finding of non-advanced adenoma and advanced neoplasia were significant risk factors for APN. Hyperplastic polyps in distal colon had an OR of 1.6 (0.7 to 3.6) and did not reach significance in this group (Table 3). From the multivariate analyses, the ORs (95% CI) of male gender, age (66–75), and distal finding of advanced neoplasia were 1.8(1.2–2.9), 4.7 (2.1–10.6), and 20.3 (12.4–33.4), respectively.

### Development of the risk score

Each risk factor was weighted in points according to the respective adjusted OR from binary logistic regression analysis (Table 3). The respective adjusted OR was halved and rounded to the nearest whole number^14^. The new index was generated assigning points to each risk factor for APN, according to age, sex and distal findings (Table 4).

The new index has a range of 0 to 13 points based on a summation of scores from each variable. The frequency distribution of subjects by new index is shown in Table 5. The cut-off value was determined by analysis of the sensitivity, specificity, positive predictive value and positive likelihood ratios for each possible score (Table 6). As a screening test, a higher sensitivity will be required. We divided the derivation cohort into two groups of risk: score 0 to 2 as “average risk” (AR); score > 2 as “high risk” (HR). Based on this stratification, 2,986 subjects (76.8%) were in the AR group and 903 subjects (23.2%) in the HR group among the derivation cohort (Table 7). The APN rates in AR and HR group were 1.2% (95% CI, 0.8–1.6%) and 7.6% (95% CI, 6.0–9.6%), respectively. The AUC in derivation cohort was 0.801 (95% CI, 0.754–0.847).

In validation cohort, the APN rates in AR and HR group were 1.5% (95% CI, 1.0–2.3%) and 5.5% (95% CI, 3.6–8.1%), respectively (Table 6). Those in the HR group had a 3.57-fold risk of having APN comparing with those in AR group (P < 0.001). The number needed to screen for one APN in HR group was 18 (95% CI, 12–28). The AUC for the new index was 0.722 (95% CI, 0.649–0.794), indicating good discrimination.

## Discussion

Risk stratification for average risk population in CRC screening offers the potential to improve the cost-effectiveness of a screening program, especially for deprived regions with limited resources^11^,^15^,^16^. Moreover, risk assessment tools for risk stratification provide tailoring options for individuals, this facilitating their decision making in the choice of the screening test^14^,^17^,^18^. Our study focused on prediction of APN, which could serve as an algorithm for determining colonoscopy referral among those who have received FS.

There are several risk algorithms devised for prediction of APN^13^,^15^,^19^,^20^,^21^,^22^,^23^,^24^,^25^. These risk models used different criteria to estimate the risk of APN. Some used distal findings alone whilst others incorporated additional variables with an intention to enhance the score’s discriminatory capability. The possible drawback of the latter types of algorithms includes the practical difficulties for them to be applied in clinical practice. Hence, the present risk score included only three variables that may facilitate its use in real-life screening services.

In our previous study^19^, we tested the external validation of Imperiale *et al*. model in the total 5,833 and obtained a result of area under curve (AUC) 0.724 (95% CI, 0.685–0.763). We also found that age, gender, and distal colon findings were all independent predictors of APN. Consistent with the previous studies^20^,^21^,^22^, our data showed that distal adenomas, but not distal hyperplastic polyps, were associated with increased risk for APN. Moreover, distal neoplasia, including advanced neoplasia and non-advanced adenoma, are consistently found to be risk factors of APN^23^. The OR of distal advanced neoplasia for APN was 2.7–3.6 when compared with normal distal findings^24^,^25^. However, in our study, our adjusted OR for APN conferred by distal advanced findings in multivariate logistic regression was relatively high (20.3, 95% CI, 12.4–33.4), which may be due to the high proportion of older people included in the present cohort. Participants with age over 60 consist of 50.5% of the derivation cohort. It is commonly recognized that distal neoplasia detected by FS, including adenoma and advanced neoplasia, should be referred for colonoscopy follow-up^4^,^26^. In our study (Table 7), the APN rate in the AR group was lower than the overall rate. It indicates that women aged 50 to 65 and men aged 50 to 55 who had non-advanced adenoma in the distal colon may not be a top priority for follow-up colonoscopy. Soon *et al*. suggested arranging colonoscopy for patients with distal advanced neoplasia to optimize colonoscopy efficiency and yield in Chinese communities with limited health-care resources^27^. Future prospective studies are needed to further quantify the risk of APN in different Chinese populations.

Our study has several strengths. First, this was the first study that has used distal finding as a predictor for predicting APN in Chinese populations. Second, we included a large cohort of asymptomatic average-risk individuals and this enables application of this model in future population-based programs. Also, the histopathology examinations were performed by the same team of pathologists who were blinded to this study to minimize potential biases. Finally we restricted our analyses to those with good and excellent bowel preparations that allowed complete observation of the whole colorectum to avoid misclassification.

There are several limitations that should be addressed. Firstly, the sampling methodology in the large hospital-based endoscopy unit was not random. Although the quality of endoscopic procedure could be standardized according to guidelines, the socio-demographic details of the subjects could be different from that of the general population. Also, the distal findings for APN prediction were derived from colonoscopy rather than FS, which the present study aims to simulate. In addition, other potential risk factors were not available in this study, such as family history, body mass index, and smoking status. This requires cautions in interpretation of the findings. Theoretically, applying more predictors will optimize the scoring model with higher AUC^28^ although too many factors might limit its practical application. Despite all the limitations, our data showed good discrimination from the new scoring index in this Chinese population.

Recently, the prognosis of proximal colon cancer continues to be poor as this was first reported since 1980 in Switzerland^29^. In usual clinical practice, proximal colon cancer had a miss rate of 4.0% during colonoscopy test^30^. It could be caused by the smaller size of advanced proximal neoplasia compared with distal one^31^,^32^. Thus, the present APN prediction model could also inform colonoscopists to raise alert not to miss proximal lesion when they encounter distal polyps during the test.

## Conclusion

The new clinical index is a useful model to stratify risk for APN in Chinese population. A FS-based risk stratification strategy is theoretically appealing, because the screening tests for lower risk individuals are less invasive, while colonoscopy could be reserved for those at higher risk. We recommend this scoring system should be externally validated in other population groups, and economic analysis be performed to study its cost-effectiveness.

## Methods

### Design

From January 2013 to December 2015, 11,554 colonoscopies were consecutively performed in a large endoscopic center of Ruijin Hospital North, Shanghai Jiaotong University. We recruited subjects who were referred by outpatient clinics and health assessment units of a major hospital, as well as screening participants under the government CRC screening program for colonoscopy. Individuals with visible bloody or abnormal stool, acute abdominal pain, abdominal mass, and the presence of CRC symptoms^33^ were excluded. CRC symptoms refer to haematochezia, malena, anorexia or a change in bowel habit in the past 4 weeks, or a weight loss of greater than 5 kg in the past 6 months. Participants from population-based CRC screening programs were all positive in immunochemical fecal occult blood tests (Kaichuang test strip (Nation Permission Number S20043085), 100 ng/ml as cut off value) by screening strategy. Other exclusion criteria included those who failed to reach the cecum (n = 329), had poor bowel preparation (n = 124), had previous history of colorectal cancer (n = 378), were diagnosed as having ulcerative colitis and Crohn’s disease by endoscopy (n = 232), were diagnosed of other colorectal disease, like familial adenomatous polyposis, melanosis coli, or colic bleeding (n = 113) and took a surveillance colonoscopy (n = 743). Another 3,801 cases were excluded because they did not reach our recommended age of screening (50–75 years old). Finally, there were 5,833 eligible cases enrolled in the study. All methods were performed in accordance with the relevant guidelines and regulations. The collection and use of clinical data was approved by the Research Ethics Committee of Ruijing Hospital North. Written informed consent was obtained from all patients before case enrollment.

### Study Procedures, Definitions

Polyethylene glycol lavage solution was used for bowel preparation. Colonoscopy was performed by experienced endoscopists using a standard video colonoscopy. Complete colonoscopy is defined as intubation of the cecum with photo documentation of cecal landmarks. Participants with incomplete colonoscopy were excluded from the analysis. The size of a polyp was estimated by open-biopsy forceps before polypectomy was performed. The methods for assessing location were chosen by the endoscopist. Lesions located in the rectum, sigmoid, or descending colon were classified as distal, whereas those located in the splenic flexure, transverse colon, hepatic flexure, ascending colon, or cecum were classified as proximal. All polyps removed during colonoscopy were sent for histologic examination. Polyps considered too large for polypectomy and other suspicious lesions were biopsied. Histologic specimens are reviewed by a team of expert pathologists who are unaware of colonoscopy findings throughout the study. The reporting of histology for colorectal neoplasms is classified according to the criteria from the World Health Organization^34^. Advanced neoplasia was defined as invasive cancer, an adenoma sized at 10 mm or more, any lesions with at least 25% villous components, an adenoma with high-grade dysplasia, or cancer. Individuals with a pathologic interpretation of carcinoma *in situ* were classified as subjects with high-grade dysplasia. If either or both the proximal or distal colon had more than one polyp, the colonic lesion was categorized according to the most advanced finding.

### Division plan and characteristics of derivation cohort and validation cohort

Based on this previous study, we randomly divided all eligible subjects into a derivation and validation cohort with a 2:1 ratio. Regarding the sample size of the validation cohort, we made reference to the study by Yeoh *et al*. in Asian countries, which indicated a minimum of 1,800 cases to attain a power of 80% for detection of a risk factor with OR of 2 at an alpha level of 0.05. This is based on the estimated prevalence of advanced neoplasia of 4.5% in other Asian colorectal advanced neoplasia studies^14^,^33^,^35^.

### Development of risk score from the derivation cohort

Univariate analysis was performed on the derivation cohort to examine the association between clinical risk factors, neoplasia, and advanced neoplasia. Variables associated with advanced neoplasia in univariate analyses (p < 0.05) were included in multivariate logistic regression analysis. For each risk factor, we assigned weightings in the risk score by using the respective adjusted ORs from the logistic regression analysis. The latter was halved and rounded to the nearest whole number to keep the total score as simple as possible. The risk score for an individual was the summation of their individual risk factors. The validity of the score and cut-off value were assessed by an AUC analysis.

### Calculation and validation of the risk score (in validation cohort)

Each subject in the validation group received a score which was the summation of individual scores based on the new index from the derivation cohort. According to the prevalence of APN in different scores, we separated the validation cohort into high risk (HR) and average risk (AR) group. The discrimination ability of the new index was examined by the c-statistics and the relative risk (RR) of APN in the HR group (versus AR group). The performance of the new index was evaluated by the AUC for prediction of APN.

### Statistical analysis

The Cochran Mantel–Haenszel χ^2^ tests were used for categorical data to compare proportions of each candidate risk factor, including age, gender, and the most advanced distal findings. Univariate and multivariate binary logistic regression were applied to assess these predictors for APN. An AUC of >0.8 and 0.7–0.8 was considered to demonstrate excellent and good discriminatory performance, respectively. Significance was defined at the P < 0.05 level for all analysis. The 95% confidential interval (CI) was reported for all the proportions. The Statistical Package for Social Science (SPSS) version 21.0 (Chicago, Illinois) was used for data analysis.

## Additional Information

**How to cite this article:** Liwen Huang, J. *et al*. An algorithm to predict advanced proximal colorectal neoplasia in Chinese asymptomatic population. *Sci. Rep.* **7**, 46493; doi: 10.1038/srep46493 (2017).

**Publisher's note:** Springer Nature remains neutral with regard to jurisdictional claims in published maps and institutional affiliations.

## Figures and Tables

**Table 1:** Characteristics of participants in the derivation and validation cohorts.

	Derivation cohort N = 3,889	Validation cohort N = 1,944	p-value
Age (years), mean ± SD	60.1 ± 6.3	60.1 ± 6.2	0.775
Gender, male, N (%)	1,814 (46.6)	912 (46.9)	0.846
Most Advanced Distal Findings, n(%)			0.725
No polyp	2,705 (69.6)	1,365 (70.2)	
Hyperplastic	362 (9.3)	176 (9.1)	
Non-advanced Adenoma	642 (16.5)	325 (16.7)	
Advanced Neoplasia	180 (4.6)	78 (4.0)	

**Table 2:** Prevalence of advanced proximal neoplasia in the derivation cohort by risk factors.

	All subjects	APN, n = 104	
	Prevalence (%)	Prevalence (%)	p-value
Gender			<0.001
Male	1,814 (46.6)	71 (3.9)	
Female	2,075 (53.4)	33 (1.6)	
Age			<0.001
50–55	862 (22.2)	7 (0.8)	
56–60	1,066 (27.4)	23 (2.2)	
61–65	975 (25.1)	27 (2.8)	
66–75	986 (25.4)	47 (4.8)	
Most Advanced Distal Findings			<0.001
No polyp	2,705 (69.6)	33 (1.2)	
Hyperplastic	362 (9.3)	7 (1.9)	
Non-advanced	642 (16.5)	21 (3.3)	
Advanced	180 (4.6)	43 (23.9)	

APN: Advanced Proximal Neoplasia.

**Table 3:** Univariate and multivariate predictors of advanced proximal neoplasia in the derivation cohort.

Unadjusted			Adjusted			
Risk factors	OR (95% CI)	p-value	βcoefficient	SE	OR (95% CI)	p-value
Gender		<0.001				0.008
Female	Referent				Referent	
Male	2.5 (1.7–3.8)		0.604	0.227	1.8 (1.2–2.9)	
Age		<0.001				0.001
50–55	Referent				Referent	
56–60	2.7 (1.2–6.3)	0.022	0.914	0.443	2.5 (1.0–5.9)	0.039
61–65	3.5 (1.5–8.0)	0.003	1.037	0.436	2.8 (1.2–6.6)	0.017
66–75	6.1 (2.7–13.6)	<0.001	1.545	0.417	4.7 (2.1–10.6)	<0.001
Most advanced distal findings		<0.001				<0.001
No polyp	Referent				Referent	
Hyperplastic	1.6 (0.7–3.6)	0.265	0.344	0.423	1.4 (0.6–3.2)	0.417
Non-advanced	2.7 (1.6–4.8)	<0.001	0.800	0.289	2.2 (1.3–3.9)	0.006
Advanced	25.4 (15.6–41.3)	<0.001	3.011	0.253	20.3 (12.4–33.4)	<0.001

**Table 4:** Scoring System for the Risk Index of Advanced Proximal Neoplasia.

Risk factor	Criteria	Points
Age	50–55	0
	56–65	1
	66–75	2
Gender	Female	0
	Male	1
Most Advanced Distal Findings	No polyp	0
	Hyperplastic polyp	0
	Non-advanced adenoma	1
	Advanced neoplasia	10

^*^A score is generated by adding points for age, gender, and most advanced distal findings.

**Table 5:** Risk for APN in the derivation cohort, by Index Score.

Score	Subjects (Proportion of Cohort) with Score, n(%)	Subjects with Score who had APN, n (%)	Risk Tier
0	428 (11.0)	2 (0.5)	Average Risk
1	1,278 (32.9)	7 (0.5)	
2	1,280 (32.9)	26 (2.0)	

3	597 (15.4)	20 (3.4)	High Risk
4	126 (3.2)	6 (4.8)	
10	9 (0.2)	0 (0.0)	
11	46 (1.2)	7 (15.2)	
12	85 (2.2)	22 (25.9)	
13	40 (1.0)	14 (35.0)	

APN: Advanced Proximal Neoplasia.

**Table 6:** Performance among different cut-off values in the derivation cohort.

Cutoff Value	Sensitivity (%)	Specificity (%)	PPV(%)	PLR
0	98.1	11.3	2.9	1.1
1	91.3	44.8	4.4	1.7
2	66.3	78.0	7.6	3.0
3	47.1	93.2	16.0	6.9
4	41.3	96.4	23.9	11.4

PPV: Positive Predictive Value; PLR: Positive Likelihood Ratios.

**Table 7:** Risk for APN, by Risk Group.

Derivation cohort			Validation cohort		
Risk Tier (Risk Score)	Subjects with score, n(%)	Subjects with score and APN, n (%) (95% CI)	Subjects with score, n(%)	Subjects with score and APN, n (%) (95% CI)	Relative Risk (95% CI)
Average Risk (0–2)	2,986 (76.8)	35 (1.2) (0.8–1.6)	1,504 (77.4)	23 (1.5) (1.0–2.3)	1.00
High Risk ( >2)	903 (23.2)	69 (7.6) (6.0–9.6)	440 (22.6)	24 (5.5) (3.6–8.1)	3.57 (2.03–6.26) P < 0.001
Total	3,889 (100)	104 (2.7) (2.2–3.2)	1,944 (100)	47 (2.4) (1.8–3.2)	

APN: advanced proximal neoplasia, CI: confidence Interval.
